# Analysis of the gap in PCR monitoring availability for patients with chronic myeloid leukemia in 60 low- and middle-income countries

**DOI:** 10.1186/s12962-021-00271-x

**Published:** 2021-03-12

**Authors:** Seth Rowley, Pat Garcia-Gonzalez, Jerald P. Radich, Ann Kim Novakowski, Irina Usherenko, Joseph B. Babigumira

**Affiliations:** 1 Department of Epidemiology, School of Public Health, University of Washington, Seattle, USA; 2 The Max Foundation, Seattle, WA USA; 3 Fred Hutchinson Cancer Research Center, Seattle, WA USA; 4 Global Medicines Program, Department of Global Health, School of Public Health, University of Washington, Seattle, USA; 5 The Comparative Health Outcomes, Policy, and Economics (CHOICE) Institute, University of Washington, Seattle, USA

## Abstract

**Purpose:**

To estimate the resource gap in the polymerase chain reaction (PCR) monitoring for patients with chronic myeloid leukemia (CML) in low- and middle-income countries (LMICs).

**Methods:**

We developed a model of demand and supply of PCR monitoring of CML patients in 60 LMICs. PCR testing was assumed to use Cepheid’s GeneXpert® ^IV^ system. We included costs of GeneXpert® instruments, uninterrupted power supplies, warranties, calibration kits, test cartridges, and shipping. We calculated the country-specific monetary gap in PCR monitoring, stratified by country priority defined as the availability of tyrosine kinase inhibitors (TKIs) through The Max Foundation initiatives.

**Results:**

The 5-year gap in PCR monitoring was $29.1 million across all countries, 22% ($6.4 million) in countries with all five TKIs available, 20% ($5.7 million) in countries with four TKIs available, 50% ($14.5 million) in countries with three TKIs available, 8% ($2.2 million) in countries with two TKIs available, and 1% ($0.3 million) in countries with one TKI available. The gap was highest in South Asia (52%; $15.1 million) and lowest in Latin America (6%; $1.9 million). Excluding labor costs, the bulk of the resource needs (86%; $25.2 million) were for procurement of BCR-ABL cartridges.

**Conclusion:**

Removing the 5-year gap in PCR monitoring capacity for CML in LMICs will require the mobilization of significant resources and will likely lead to better treatment outcomes and reduced treatment costs through optimization of treatment, discontinuation of therapy in appropriate patients, and facilitation of clinical research. Development of streamlined monitoring guidelines for resource-limited countries should be considered.

## Background

The advent of tyrosine kinase inhibitors (TKIs) has transformed chronic myeloid leukemia (CML) from a near universally fatal disease into a chronic condition. There are now five TKIs approved for use in newly diagnosed CML in the USA, the pioneering TKI imatinib, and the “second generation” TKIs dasatinib, nilotinib, ponatinib, and bosutinib. All have similarly impressive results and CML patients now experience life expectancy near that of the age matched general population [1].

CML is caused by the genetic juxtaposition of the BCR gene from chromosome 22 with the tyrosine kinase domains of the ABL gene from chromosome 9. This unique gene fusion creates the fusion BCR-ABL1 mRNA, which is the target for polymerase chain reaction (PCR) diagnostic and monitoring assays. The protein product of BCR-ABL translocation drives the pathogenesis of CML and is the target of the TKIs.

Peripheral blood can be used for BCR-ABL monitoring, rather than bone marrow, and guidelines of the European Leukemia Network (ELN) and the National Cancer Care Network (NCCN) include frequent monitoring as a way to track response. In general, both guidelines call for every 3-month peripheral blood PCR testing for BCR-ABL1 [2, 3]. Treatment milestones, including changing TKIs for resistance, or discontinuation of TKIs after a durable deep response, are based on the BCR-ABL1 monitoring.

Conventional PCR testing is complicated and labor intensive, demanding trained personnel, many chemicals, and expensive equipment. Quality control is essential to obtain and maintain proper sensitivity and specificity of the assay, and this requires technical and methodological expertise and training. The GeneXpert system from Cepheid is a cartridge-based system that requires minimal hands on sample preparation and is ideal for point of care, real-time assays [4]. The system has a multiple modular design that can run cartridges for multiple diseases including infections and cancer. The assay for BCR-ABL1 was developed in collaboration with U.S. academic centers and is now used in many centers in the U.S. and Europe for CML monitoring in clinical trials and community settings, and is FDA approved in the U.S. The system is widely used in low- and middle-income countries (LMICs) for infectious diseases (e.g., TB, HIV) and utilization is increasing in a handful of oncology applications including BCR-ABL1 [5, 6]. The Cepheid platform has many desired features in the LMIC setting, including ease and efficiency of use (less technical training needed, with flexible use between batches or single assays), easy shipping solutions (cartridges rather than many different reagent supplies), high reproducibility, and reliable precision and accuracy.

To bring TKIs to LMICs, The Max Foundation partnered with Novartis to launch the Glivec® International Patient Assistance Program (GIPAP), an innovative access model that made Novartis’ breakthrough oral TKI therapy, Glivec® (imatinib) available to patients at no cost. Glivec was made available to patients in selected LMICs around the world who met specific program criteria, such as confirmed indication for the medication, presence of partner institutions for diagnosis and management, and tax waivers for donated TKIs by governments among other criteria [7]. The Max Foundation administered the program, working closely with a global network of more than 1500 trained physicians in 80 LMICs [7].

In the early years of the program, BCR-ABL1 testing was largely performed by shipping blood to outside centers, a procedure that was costly, time-consuming, and inherently unscalable. Recognizing the critically important role that point-of-care systems play in the diagnosis and monitoring of patients in resource poor settings, The Max Foundation developed a collaboration with Cepheid to increase access to instruments and tests in more than 60 LMICs. Cepheid provided the GeneXpert® System and Xpert® BCR-ABL Monitor Assays (through The Max Foundation) to public sector end users at a preferential price in order to decrease barriers to access due to cost. The dramatic increases in monitoring meant disease resistance could be detected, so that TKI options to treat resistance could be initiated as needed. The Max Foundation began partnering with four other leading pharmaceutical companies to bridge access to all five TKIs available on the market for the treatment of CML today. As a result of these efforts, selected patients in thirty of the world’s lowest income economies have access to all available treatment options through The Max Foundation’s Max Access Solutions.

Recently, GIPAP evolved into a new partnership model between Novartis and The Max Foundation, CMLPath to Care™. CMLPath to Care™ differs from the GIPAP program in that The Max Foundation assumes from Novartis the responsibility for delivering treatment to patients. The Max Foundation, under the umbrella of Max Access Solutions (MAS), manages the entire supply chain, while strengthening interactions with local stakeholders and providing hands-on, local patient support [8].

The establishment of collaborations with developers of all TKIs for the treatment of CML enables The Max Foundation to respond to health care providers’ requests for second and third line treatment in many of the lowest income economies in the world. Yet, despite the preferential pricing available for Cepheid products, access to PCR remains one of the most pervasive barriers to achieving optimal clinical outcomes. Without adequate monitoring, physicians are unable to make timely and appropriate clinical decisions that help patients with CML achieve a deep response to available treatments. This analysis was performed to assess the resources needed to close the 5-year gap in PCR monitoring capacity for CML patients covered by programs supported by the Max Foundation in countries where The Max Foundation currently operates.

## Methods

### Analytic overview

We developed a Microsoft Excel-based demand, supply, and forecast model for the monitoring of CML patients in a selected group of countries and a selected group of patients in those countries covered by access initiatives and using preferential prices available through The Max Foundation for the GeneXpert® system. The gap in PCR capacity for monitoring was estimated as the difference between current/projected demand and current/projected supply over a 5-year time horizon from 2017 to 2021.

We estimated effective demand as the number of patients that would qualify for access to imatinib treatment in each country i.e. patients who would qualify for treatment under The Max Foundation’s access initiatives. This is differentiated from actual demand based on the burden of CML in each country, a burden that is substantially higher than is covered by The Max Foundation’s access initiatives [9]. We estimated supply as the number of patients that are able to access at least 3 PCR tests per year, in line with monitoring recommendations [2, 3]. We included an additional 0.5 tests per individual per year to account for off-label use of the Xpert® cartridges for diagnostic purposes, as we are aware that physicians in resource poor settings may utilize the cartridges to confirm presence of BCR-ABL, thereby meeting medical eligibility requirements for treatment access. Therefore, the supply estimate was based on the number of patients able to access 3.5 tests per year.

Countries were categorized for the analysis by availability of one or more of the five TKIs to maximize the potential utility of PCR monitoring results by physicians. Countries were excluded from the analysis if they were known to have appropriate access to PCR and if they did not have preferential pricing for GeneXpert PCR through Cepheid.

### Demand estimation

We estimated future demand for PCR monitoring in The Max Foundation programs based on historical patient data from The Max Foundation. We restricted our demand estimates to patients who would qualify for Max Access Solutions and The Max Foundation’s preferential pricing agreement with Cepheid. Of the 67 countries covered by Max Access Solutions and The Max Foundation’s preferential pricing agreement with Cepheid, 56 had 12 years of historical data, four countries had 11 years of historical data, two countries had 10 years of historical data, three countries had 8 years of historical data, one country had 7 years of historical data, and one country had 4 years of historical data. In our final analysis, we included 60 countries after excluding Angola, Barbados, Botswana, Costa Rica, Guinea, Pakistan, and Panama because they did not have patients currently receiving treatment through The Max Foundation programs. For countries with non-linear historical trends, the earlier, nonlinear years were truncated from the analysis. Estimates of future demand, estimated as number of patients, were calculated using a simple linear ordinary-least-squares regression.

Data from The Max Foundation demonstrate that the introduction of GeneXpert® affects the demand for CML treatment, and consequently, the demand for PCR monitoring in a country. Therefore, based on consensus among the study team members, we made the assumption and modeled that the rate of growth doubled (2x) after introduction of GeneXpert®, e.g., a country that historically gained 10 additional patients per year before GeneXpert® introduction, was modeled, after introduction, as gaining 20 additional patients per year.

### Estimation of the gap in PCR monitoring capacity

The quantity of required GeneXpert® instruments and GeneXpert® cartridges for purposes of gap estimation was based on a number of factors: current demand, anticipated future demand, current number of GeneXpert® instruments in place by country, geographic demand distribution of patients and testing facilities, and expert opinion in the form of NCCN and ELN guidelines, as described above.

### Costs

We estimated the following PCR monitoring-related costs in year 1: (1) GeneXpert® instruments, (2) uninterrupted power supplies (UPS), (3) warranties, (4) calibration kits, (5) test cartridges, and (6) shipping for instruments and test cartridges. During years two to five, the following PCR monitoring-related costs were included: (1) calibration kits, (2) test cartridges, and (3) shipping costs for cartridges. Costs did not include labor to perform the assay. Costs were obtained from The Max Foundation records and estimated in 2017 $US. Future costs were discounted to 2017 $US at a rate of 3%.

Our analysis differentiated between fixed costs in the first year (GeneXpert® instruments, UPSs, and instrument warranty) and variable costs (cartridges, shipping, and calibration). Costs for the instruments, warranty, calibration kits, and UPSs, were established by contract (see Table 1). Shipping costs were based on per-country historical shipping data when available and based on a global average when unavailable. Calibration kits are required every 2000 tests, or once per year, whichever occurs first. Instruments have a factory warranty of 2 years; additional warranty costs are included in the model for years three through five.

**Table 1:** Cost parameters*

Variable	Base Value	Normal distribution values		
		Mean	Lower	Upper
Glivec pills (per year)	365	365	182.5	547.5
Patient visits per year	3.5	3.5	1.75	5.25
GeneXpert®	$17,000	$17,000	$17,000	$17,000
Uninterrupted Power Supply	$780	$780	$390	$1,170
GeneXpert® cartridges	$50	$50	$25	$75
GeneXpert® warranty (yrs. 3–5)	$2,900	$2,900	$1,450	$4,350
GeneXpert® calibration kit	$450	$450	$225	$675
Annual Patient Growth Rate**	2x	2x	1x	3x

^*^All cost data were obtained from The Max Foundation records

^**^As a consequence of increased access to PCR monitoring using GeneXpert®

### Credibility Intervals

We constructed credibility intervals for the mean total 5-year gap cost for each country by iteratively drawing single values from a normal distribution for each component cost element (e.g. GeneXpert® cartridges), calculating the resulting mean of many draws, and then summing each component average into an overall cost for each country for each year. The means of the normal distributions from which we drew were set equal to the deterministic cost values. The upper and lower 95% intervals were set to ± 50% of the means. The presented estimates were derived from 1,000 iterations.

## Results

The total 5-year gap in PCR monitoring capacity for each country, categorized by TKI access, is presented in Table 2. Over the 5-year period, the estimated gap in PCR monitoring capacity was $29,143,083 across all countries covered by access initiatives. Over the 5-year period, due to the combination of country-specific trends and anticipated patient increases due to improved access to PCR testing, the patient population was estimated to grow from 26,915 patients to 30,071 patients at year-end 2021.

**Table 2:** Total 5-year gap

Country	Region	TKIs	Total 5-year Gap	95% CI
Benin	Africa/Middle East	5	$88,758	$86,510 to $89,150
Bhutan	Asia Pacific	5	$49,031	$48,250 to $49,160
Burkina Faso	Africa/Middle East	5	$92,401	$90,060 to $92,870
Cambodia	Asia Pacific	5	$108,594	$104,680 to $109,390
East Timor	Asia Pacific	5	$36,976	$36,590 to $37,050
Ghana	Africa/Middle East	5	$200,603	$192,860 to $201,580
Haiti	Latin America (LATAM)	5	$50,842	$50,040 to $51,040
Honduras	Latin America (LATAM)	5	$284,589	$273,850 to $286,210
Kyrgyzstan	Europe/Central Asia	5	$272,886	$263,750 to $274,330
Madagascar	Africa/Middle East	5	$98,186	$95,700 to $98,750
Malawi	Africa/Middle East	5	$70,677	$69,080 to $71,040
Moldova	Europe/Central Asia	5	$162,785	$157,760 to $163,700
Mongolia	Europe/Central Asia	5	$123,690	$119,890 to $124,130
Nepal	South Asia	5	$1,561,298	$1,506,040 to $1,571,720
Nicaragua	Latin America (LATAM)	5	$161,998	$157,000 to $162,790
Niger	Africa/Middle East	5	$58,210	$57,040 to $58,370
Nigeria	Africa/Middle East	5	$1,173,867	$1,131,180 to $1,183,210
Papua New Guinea	Asia Pacific	5	$90,538	$88,360 to $91,170
Republic of Congo	Africa/Middle East	5	$93,545	$91,130 to $93,990
Rwanda	Africa/Middle East	5	$102,710	$98,830 to $103,250
Sierra Leone	Africa/Middle East	5	$31,681	$31,490 to $31,830
Tajikistan	Europe/Central Asia	5	$73,353	$71,290 to $73,330
Togo	Africa/Middle East	5	$65,240	$63,630 to $65,460
Uzbekistan	Europe/Central Asia	5	$1,385,716	$1,336,100 to $1,395,670
Cameroon	Africa/Middle East	4	$164,268	$159,150 to $165,090
Central Africa Republic	Africa/Middle East	4	$29,515	$29,210 to $29,530
Cote d'Ivoire	Africa/Middle East	4	$207,340	$200,920 to $208,790
Democratic Republic of Congo	Africa/Middle East	4	$40,534	$40,100 to $40,700
Ethiopia	Africa/Middle East	4	$964,173	$926,070 to $969,910
Georgia	Europe/Central Asia	4	$375,196	$362,310 to $377,190
Kenya	Africa/Middle East	4	$1,102,988	$1,063,540 to $1,110,340
Mali	Africa/Middle East	4	$129,666	$125,850 to $130,250
Philippines	Asia Pacific	4	$177,153	$172,700 to $178,080
Senegal	Africa/Middle East	4	$247,913	$238,460 to $249,220
Sri Lanka	South Asia	4	$284,646	$273,970 to $286,080
Sudan	Africa/Middle East	4	$1,363,279	$1,313,480 to $1,371,390
Tanzania	Africa/Middle East	4	$197,892	$191,470 to $199,000
Uganda	Africa/Middle East	4	$339,760	$328,270 to $341,910
Zambia	Africa/Middle East	4	$98,688	$95,950 to $99,020
Bolivia	Latin America (LATAM)	3	$302,636	$293,390 to $304,270
El Salvador	Latin America (LATAM)	3	$210,811	$203,830 to $211,630
Guatemala	Latin America (LATAM)	3	$339,146	$327,800 to $341,310
India	South Asia	3	$13,238,674	$12,849,210 to $13,457,480
Paraguay	Latin America (LATAM)	3	$146,770	$142,670 to $147,700
Seychelles	Africa/Middle East	3	$31,144	$30,960 to $31,290
Surinam	Latin America (LATAM)	3	$51,723	$50,890 to $51,920
Zimbabwe	Africa/Middle East	3	$172,320	$166,040 to $173,410
Azerbaijan	Europe/Central Asia	2	$476,120	$461,130 to $481,150
Bahamas	Latin America (LATAM)	2	$35,980	$35,690 to $36,110
Belarus	Europe/Central Asia	2	$47,129	$46,400 to $47,220
Fiji	Asia Pacific	2	$53,660	$52,710 to $53,820
Jamaica	Latin America (LATAM)	2	$112,265	$109,310 to $112,920
Kazakhstan	Europe/Central Asia	2	$38,083	$39,390 to $40,380
Saint Lucia	Latin America (LATAM)	2	$41,084	$40,650 to $41,270
Vietnam	Asia Pacific	2	$1,422,762	$1,372,220 to $1,435,050
Dominican Republic	Latin America (LATAM)	1	$53,976	$51,800 to $54,040
Ecuador	Latin America (LATAM)	1	$36,238	$36,010 to $36,460
Gabon	Africa/Middle East	1	$68,943	$67,410 to $69,210
Namibia	Africa/Middle East	1	$49,909	$49,130 to $50,110
Peru	Latin America (LATAM)	1	$52,524	$51,740 to $52,860

The distribution of the total PCR monitoring capacity gap by TKI access level (Table 3) was as follows: 22.09% ($6,438,176) in countries with all five TKIs, 19.64% ($5,723,011) in countries with four TKIs, 49.73% ($14,493,223) in countries with three TKIs, 7.64% ($2,227,083) in countries with two TKIs, and 0.90% ($261,590) in countries with one TKI.

**Table 3:** Aggregate costs by region and TKI count

Region	5-year cost (%)	TKI access	First year fix	First year var	5-year cost
South Asia	$15,084,618 (52%)	5	$540,850	$909,370	$6,438,176
Africa/Middle East	$7,284,210 (25%)	4	$323,610	$865,947	$5,723,011
Europe/Central Asia	$2,954,959 (10%)	3	$579,310	$3,031,829	$14,493,223
Asia Pacific	$1,938,714 (7%)	2	$188,510	$308,242	$2,227,083
Latin America (LATAM)	$1,880,582 (6%)	1	$161,580	$32,770	$261,590
Total	$29,143,083	Total	$1,740,000	$5,148,158	$29,143,083

In those countries where all five TKIs are currently available through The Max Foundation programs, the 5-year gap ranged from $31,681 in Sierra Leone to $1,561,298 in Nepal. In the countries with four TKIs available, the gap ranged from $29,515 in Central Africa Republic to $1,363,279 in Sudan. In the countries with three TKIs available, the gap ranged from $31,144 in Seychelles to $13,238,674 in India. In the countries with two TKIs available, the gap ranged from $35,980 in The Bahamas to $1,422,762 in Vietnam. In the countries with a single TKI available through Max programs, the gap ranged from $36,238 in Ecuador to $68,943 in Gabon.

The distribution of the total PCR monitoring capacity gap by region was as follows (Table 3): 52% ($15,084,618) in South Asia, 25% ($7,284,210) in Africa/Middle East, 10% ($2,954,959) in Europe/Central Asia, 7% ($1,938,714) in Asia Pacific, and 6% ($1,880,582) in Latin America. India alone accounted for 45% ($13,238,674) of the total gap.

**Fig. 1:**
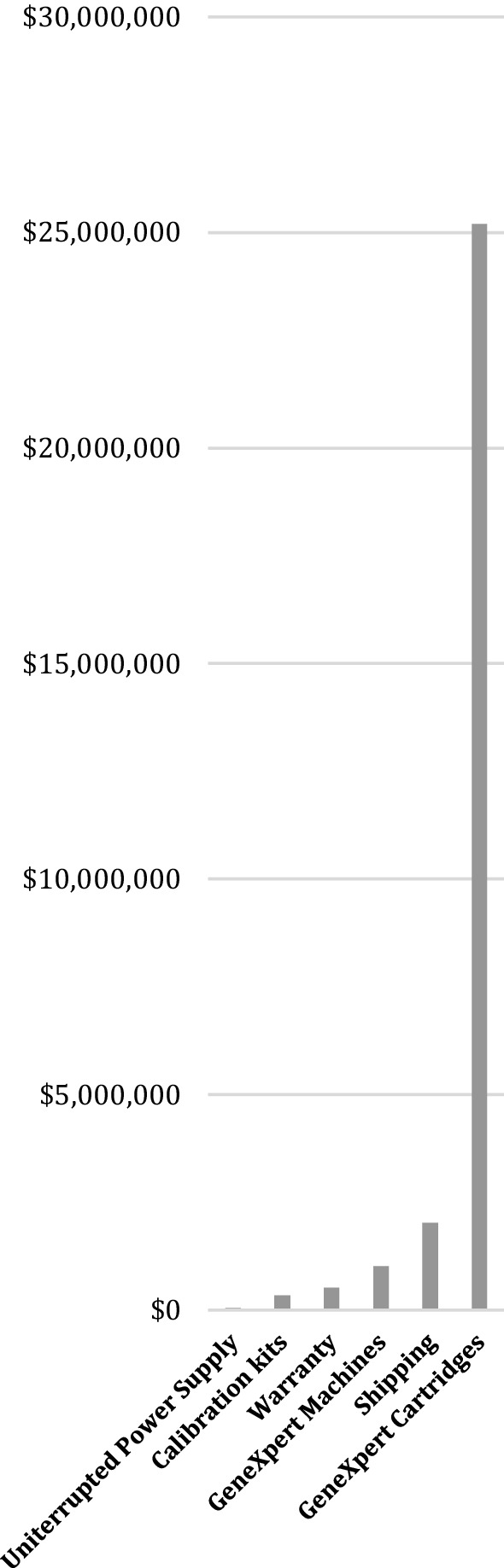
Costs by resource

The distribution of the total PCR monitoring capacity gap by resource was as follows: 86% ($25,203,261) for cartridges, 7% ($2,014,202) for shipping, 3% ($1,020,000) for GeneXpert® machines, 2% ($522,000) for warranties, 1% ($336,820) for calibration kits, and 0.2% ($46,800) for UPSs (Fig. 1).

Across the countries where The Max Foundation’s is involved, the negotiated purchase agreements with Cepheid result in a nominal cumulative cost reduction of $1,246,667 as compared with GeneXpert IV machine market pricing levels, and additional cost reductions of $30,747,979 for GeneXpert cartridges, as compared with average market prices.

## Discussion

We estimated the gap in PCR monitoring capacity for CML patients in countries covered by access initiatives available through The Max Foundation. Over a 5-year period, the total gap in PCR monitoring capacity amounts to approximately $30 million, including instrument and cartridge subsidies. This estimate corresponds to the cost of monitoring patients with CML every 3 months over a 5-year period, categorized by access to TKIs. Approximately one-fifth of the total gap in PCR monitoring capacity is in countries with access to all five TKIs through the Max Access Solutions current portfolio. One-third of the gap is in countries with access to four out of five TKIs.

There was substantial variability in the country-specific estimates of the gap in PCR monitoring capacity, based largely on the number of patients needing testing. The overall range was from $29,515 in Central African Republic to $13.2 million in India where more than 50% of The Max Foundation’s total CML patient population is treated. It would be worthwhile to apply, in addition to a TKI-access-based prioritization scheme, a prioritization scheme on a country-by-country basis to ensure that countries with GeneXpert® instruments have the resources to procure cartridges in the short- and medium-term.

Given that GeneXpert® cartridges and GeneXpert® instruments are linked resources, it is preferable to take a pragmatic approach and examine access on the basis of potential utility. For example, procuring GeneXpert® instruments to cover all countries would be inefficient, likely resulting in idle instruments due to the lack of resources to procure sufficient amounts of the BCR-ABL assay. In order to remove the PCR monitoring barrier and improve clinical care for patients with CML, it will be essential to adopt a comprehensive plan to engage partners who are vested in strengthening health systems supporting cancer care in LMIC. Drug manufacturers, non-governmental organizations, governments, interest groups, and/or philanthropists may find value in aiding specific countries to increase PCR capacity. Since the oncology portfolio for the GeneXpert® system includes testing for breast cancer, bladder cancer, and HPV, this multi-sector approach requires collaboration and coordination among agencies and organizations within LMICs to maximize partner investments and avoid the inefficiency of duplicated efforts. Moreover, investment in PCR monitoring in these countries might lead to strengthening health systems so that physicians and specialized treatment centers are able to respond to the needs of a more diverse patient population.

Nearly 90% of the overall gap in PCR monitoring capacity for CML is in the procurement of cartridges. Even at a preferential (below market) cost of $50, single-test cartridges are still costly, particularly in resource-poor settings. Although CML is a relatively rare disease worldwide, a buy-down approach consisting of an up-front, pre-purchase of Xpert® cartridges at higher volumes and reduced prices might further reduce costs. A similar approach was employed by the Bill and Melinda Gates Foundation, the United States President’s Emergency Plan for AIDS Relief (PEPFAR), the United States Agency for International Development (USAID), and Unitaid [10, 11].

A limitation of our analysis is that we used estimates of the burden of disease by country based on the number of CML patients enrolled in GIPAP and Max Access Solutions from 2002 to 2017. This is an underestimate of the true gap based on burden of disease estimates obtained from epidemiological data [9]. There are significant numbers of patients that did not enter these programs because of access limitations due to geography, culture, cost, and other social determinants of health. Future analyses might consider this epidemiological burden, combined with estimates of the gap in diagnostic and treatment (with TKIs) capacity. Ongoing research in this area can also examine to what degree the gap in CML treatment capacity is a subset of a general prevailing gap in health system capacity related to infrastructure and personnel that may not be amenable to targeted solutions such as increased access to GeneXpert® machines and supplies.

Removing the gap in PCR monitoring capacity for CML can help improve health outcomes. While quantifying the health outcomes benefit was beyond the scope of this analysis, we expect that removing the gap in PCR diagnostic capacity would increase the demand for CML care, both within the access initiatives available through The Max Foundation and in general health services. Future studies might consider quantifying the health outcomes benefits in terms of quality-adjusted life-years gained or disability-adjusted life-years averted using state-transition (Markov) models as in multiple economic evaluations of TKIs [12–16].

NCCN and ELN monitoring guidelines are based on monitoring intervals used in TKI clinical trials, and thus it is not clear what optimal testing intervals actually are. For example, optimal design might include how long a patient has been on a TKI, and the kinetics of their BCR-ABL1 decline, rather than a simplistic every 3 months rule. Thus, revisiting guidelines with limited resources in mind may allow for a CML monitoring scheduled tailored to the LMIC setting, optimizing the benefits of frequent monitoring without sacrificing patient outcomes.

## Conclusions

By treating thousands of patients since 2001 in eligible countries, The Max Foundation’s access programs have added thousands of life-years and improved the quality of life of thousands of people living with CML. Removing the 5-year gap in PCR monitoring capacity for CML in these countries will require the mobilization of significant resources and will likely lead to better treatment outcomes and reduced treatment costs through optimization of treatment, discontinuation of therapy in appropriate patients, and facilitation of clinical research.

## Data Availability

The datasets during and/or analysed during the current study, including modeling framework, available from the corresponding author on reasonable request.
